# Guided Waves Along a Density Interface in Partially Ionised Solar Plasmas

**DOI:** 10.1007/s11207-025-02576-z

**Published:** 2025-11-19

**Authors:** S. Alshammari, Istvan Ballai, Gary Verth, Viktor Fedun, Lyudmila Kozak

**Affiliations:** 1 Plasma Dynamics Group, School of Mathematical and Physical Sciences, University of Sheffield, Sheffield, S3 7RH UK; 2 Department of Mathematics, College of Science, University of Hafr Al Batin, Hafr Al Batin, 39524 Saudi Arabia; 3 Plasma Dynamics Group, School of Electrical and Electronic Engineering, University of Sheffield, Sheffield, S1 3JD UK; 4 Taras Shevchenko National University of Kyiv, 01601 Volodymyrska Street, 60, Kyiv, Ukraine; 5 Space Research Institute of the National Academy of Sciences of Ukraine and the State Space Academy of Ukraine, 03680 Glushkov Ave 40, 4/1, Kyiv, Ukraine

**Keywords:** Solar atmosphere, Waves, Guided waves, Partially ionised plasmas, Ambipolar diffusion

## Abstract

This study investigates the properties of waves that propagate along a density interface in partially ionised plasmas, separating two regions of different properties, including ionisation degree. Our analysis covers frequencies that are much smaller than the collisional frequency of particles, so we are using a single-fluid approximation, where the partial ionisation aspect of the plasma appears through the ambipolar diffusion in the generalised Ohm’s law. The derived dispersion relation is solved numerically. Our results show that guided waves along a density interface undergo very little change in their propagation speed (frequency); however, their damping rate shows variation with the ionisation degree and plasma-$\beta $ parameter. We find that waves can only propagate when plasma-$\beta >1.2$, indicating pressure-driven dynamics relevant to photospheric structures with moderate magnetic fields. The damping rate increases with higher neutral particle content but decreases with higher plasma-$\beta $ values. For ionisation degrees close to fully ionised plasma, the damping is minimal but becomes more significant as the neutral particle concentration increases. These findings provide important insights into wave behaviour in partially ionised plasma interfaces and lay the groundwork for future studies of wave propagation in partially ionised plasma slab waveguides.

## Introduction

Waves and oscillations propagating in the solar atmosphere received special attention due to their capabilities to transport and deposit energy, therefore contributing to atmospheric heating (see, e.g. Erdélyi and Ballai 2007) as well due to their use in diagnostics of the magnetic field and the dynamical and thermodynamical state of the plasma using seismological techniques (Nakariakov et al. 1999; Ballai 2007; Banerjee et al. 2007; Oliver 2009; Nakariakov et al. 2024). Very often, waves are also tracers of the magnetic field thanks to their ability to propagate along field lines. With the advancement of observational facilities, we are now able to observe and study waves and oscillations across almost the entire electromagnetic spectrum.

Various atmospheric models that were synthesised using the emission and absorption of several spectral lines predict that the electron–neutral number density ratio of the plasma in the lower solar atmosphere covers several orders of magnitude. Atmospheric models such as the VAL model (Vernazza, Avrett, and Loeser 1981), the AL model (Avrett and Loeser 2008) or the FAL model (Fontenla, Avrett, and Loeser 1993) suggest that the electron–neutral ratio in the quiet Sun ranges between $\sim 10^{6}$ to $10^{-5}$ covering distances starting at the surface of the Sun up to lower coronal heights, proving that in these regions the solar plasma is partially ionised, i.e. the plasma is a mixture of charged particles (electrons and positively charged ions) and neutrals that interact through collisions. This character of the plasma persists also in more active solar features, for example, in the case of prominences, the ratio between the electron density and neutral hydrogen density ranges between 0.1 and 10 (Patsourakos and Vial 2002).

The qualitative and quantitative description of a partially ionised plasma requires a more complex framework than the fully ionised plasma, and this stems from the necessity of describing the dynamics of each species separately as well as their coupling. Analytical progress can be obtained assuming the extreme situations of weakly and strongly ionised limits in which the ratio of ion and neutral number density (or its reciprocal) acts as a small parameter and governing equations can be expanded with respect to this quantity (Alharbi et al. 2021, 2022). The framework in which dynamics is described also depends on the frequency range we are interested in. For frequencies much lower than the collisional frequency of particles (as assumed in the present study), the plasma dynamics can be confidently described using a single-fluid approximation, in which partially ionised effects appear through the generalised Ohm’s law in the form of ambipolar diffusion that dissipates perpendicular currents to the ambient magnetic field.

In partially ionized plasma, ambipolar diffusion arises when neutrals are not fully coupled to the motion of charged components. Charged particles are influenced by the Lorentz force, whereas the neutrals move randomly due to Brownian motion. However, neutral particles still interact with ions through short-range (head-on) collisions. These interactions generate friction between the two components, providing the dissipation of magnetic and mechanical energy, and hence leading to localized atmospheric heating (see, e.g. Forteza et al. 2007; Shelyag, Mathioudakis, and Keenan 2012; Khomenko et al. 2018; Yalim et al. 2020; Popescu Braileanu and Keppens 2021; McMurdo et al. 2023).

Neutrals, as well as playing a significant role in the dissipation rates of magnetohydrodynamic (MHD) waves, also contribute to stabilizing instabilities (see, e.g., Soler et al. 2012; Díaz, Khomenko, and Collados 2014; Ballai et al. 2017; Mather, Ballai, and Erdélyi 2018; Ruderman et al. 2018; Murtas, Hillier, and Snow 2024; Snow and Hillier 2024).

Khomenko et al. (2021) investigated the role of ambipolar diffusion in three-dimensional magneto-convection simulations and observed that it significantly reduces vorticity in the upper chromosphere. This reduction occurs as ambipolar diffusion dissipates vortical disturbances, converting them into thermal energy. These findings suggest a strong link between the presence of neutrals and the effective damping of waves, which may contribute to localized heating in the partially ionized solar atmosphere. Similarly, Shelyag et al. (2016) studied the nonlinear propagation of waves in a three-dimensional stratified solar flux tube with ambipolar diffusion. Their results showed that up to 80% of the Poynting flux carried by these waves can be dissipated and transformed into heat, delivering substantially more energy to the chromosphere compared to the dissipation of stationary currents, as modelled by Khomenko and Collados (2012). Martínez-Sykora et al. (2015) also used a 2.5D radiative MHD model to demonstrate that ambipolar diffusion efficiently dissipates magnetic energy, increasing chromospheric temperature.

Tangential discontinues separating two regions of different properties are natural phenomena in solar and space plasmas. These discontinuities can support the propagation of MHD waves, similar to the waves that appear at the interface between, say, water and air. The study by Roberts (1981) showed that an interface separating two plasma regions with a magnetic field oriented along the interface can support the propagation of fast magnetoacoustic waves with a phase speed situated between the Alfvén speeds in the two regions. In addition, the study also found that if one of the regions is field-free, slow MHD waves will propagate along the interface, while if the field-free region is warmer than the magnetic region, an additional fast wave can also propagate.

Our paper aims to analyse the properties of guided waves propagating along a density interface when the discontinuity separates two partially ionised plasmas that correspond to the environment found in the lower solar atmosphere. The results obtained earlier by Roberts (1981) will serve us as a benchmark, and we will interpret our results in the light of the findings of this study. Our paper is structured as follows: in Section 2, we will derive the dispersion relation of guided waves at a single interface. The solution of this dispersion relation will be obtained numerically and discussed in Section 3. Our results are concluded and summarised in Section 4.

## Dispersion Relation of Waves Propagating Along a Sharp Boundary

In general, the determination of a dispersion relation of waves propagating in an inhomogeneous plasma (either with inhomogeneity along the direction of propagation or in the transversal direction) is an impossible task, and the possible solutions can be obtained by solving numerically evolutionary differential equations. The problem becomes tractable when the inhomogeneity is chosen to be rather specific. In our approach, we will deal with an inhomogeneity in the transversal direction to the propagation of waves, and this will be chosen to be represented by a sharp boundary between two, otherwise homogeneous, half-planes. Accordingly, we will use a Cartesian coordinate system, and the boundary between the two regions (or interface) coincides with the $yz$-plane, i.e. its equation is $x=0$.

The propagation of waves along the interface will be studied by assuming that this surface separates two regions (labelled by indices 1 and 2), which are permeated by the same magnetic field; however, the density in the two regions is different, i.e. we are dealing with a density jump. The equilibrium magnetic field is unidirectional and it is oriented in the positive $z$-direction. A consequence of the specific equilibrium we use here is that by the continuity of the total pressure (kinetic and magnetic), the two regions have the same equilibrium pressure, $p_{0}$. The constant density of the two regions is given as

1 $$\rho_{0}=\left\{ \begin{array}{ll} \rho_{1}, & x<0,\\ \rho_{2}, & x>0. \end{array}\right.$$

The plasma in the two regions will be considered to be partially ionised and the dynamics in both regions will be described by the linearised set of MHD equations given by

2 $$ \frac{\partial \rho}{\partial t} + \rho _{0}\nabla \cdot{\mathbf{v}} = 0, $$

3 $$ \rho _{0}\frac{\partial{\mathbf{v}}}{\partial t} = - \nabla p + \frac{1}{\mu _{0}}(\nabla \times{\mathbf{b}})\times{ \mathbf{B}}_{0}, $$

4 $$ \frac{\partial{\mathbf{b}}}{\partial t} = \nabla \times \left \{{\mathbf{v}} \times{\mathbf{B}}_{0} + \frac{\eta _{A}}{B_{0}^{2}}[(\nabla \times{\mathbf{b}}) \times{ \mathbf{B}}_{0}] \times{\mathbf{B}}_{0}\right \}, $$

5 $$ \frac{\partial p}{\partial t} = -\gamma p_{0}\nabla \cdot{\mathbf{v}}, $$

6 $$ \nabla \cdot \textbf{b}=0, $$

where the quantities labelled by an index 0 ($\rho _{0}$, ${\mathbf{B}}_{0}$, $p_{0}$) describe the equilibrium state, ${\mathbf{v}}=(v_{x},0,v_{z})$ and ${\mathbf{b}}=(b_{x},0,b_{z})$ are the velocity and magnetic field perturbations. The quantities $p$ and $\rho $ denote the perturbations for pressure and density. The constants $\mu _{0}$, $\gamma $ are the permittivity of free space and the adiabatic index, respectively. The equilibrium magnetic field is assumed to be homogeneous and oriented along the $z$-axis.

Partial ionisation effects appear only in Equation 4 through the ambipolar diffusion whose coefficient is $\eta _{A}$ and it is defined as (see, e.g. Forteza et al. 2007; Díaz, Khomenko, and Collados 2014; Ruderman et al. 2018; MacBride et al. 2022)

7 $$ \eta _{\mathrm{A}} = \frac{m_{\mathrm{p}}\xi _{\mathrm{n}}v_{A}^{2}}{4\sigma _{\mathrm{in}}(1-\xi _{\mathrm{n}})}\sqrt{ \frac{\pi (2-\xi _{\mathrm{n}})}{\rho _{0} p_{0}}}= \frac{m_{p}(\pi \gamma )^{1/2}}{4\sigma _{in}} \frac{\xi _{n}(2-\xi _{n})^{1/2}}{1-\xi _{n}} \frac{v_{A}^{2}}{\rho _{0}c_{S}}. $$

In the above expression $m_{\mathrm{p}}$ is the proton mass (for a hydrogen plasma this mass is approximately equal to the mass of the neutral particles), the index $n$ stands for the neutral particles, $\xi _{\mathrm{n}}=\rho _{n}/\rho _{0}\approx n_{n}/n$ is defined as the ratio of the number density of neutrals and the total number density (neutrals plus ions), $\sigma _{\mathrm{in}} \approx 5\times 10^{-19}\text{ m}^{2}$ is the constant collisional cross-section for proton–neutral collisions, $v_{A}$ is the Alfvén speed defined as $v_{A} = B_{0}/\sqrt{\mu _{0}\rho _{0}}$ and $c_{S}=\sqrt{\gamma p_{0}/\rho _{0}}$ is the adiabatic sound speed. In the above equations, $\rho _{0} = \rho _{e}+\rho _{i} +\rho _{n} \approx \rho _{i} + \rho _{n}$ is the total density (the sum of densities of electrons ($e$), ions ($i$), and neutrals ($n$)). Here, we neglected the density of electrons since their mass is much smaller than the mass of protons or neutrals. Strictly speaking, the effect of the ambipolar diffusion should have appeared in the energy equation 5, as well, through the Ohmic dissipation of perpendicular currents, but since this term is nonlinear, it will be neglected.

We can introduce the ionisation degree of the plasma, $\mu $, defined as (Ballai, Forgács-Dajka, and Marcu 2019)

$$ \mu =\frac{1}{2-\xi _{n}}. $$

Accordingly, ${\mu}=0.5$ corresponds to a fully ionised plasma, while ${\mu}=1$ corresponds to a fully neutral gas. From the definition of ${\mu}$, we can write that $\xi _{n}=2-1/\mu $. As a result, the expression of the ambipolar diffusion can be written as

8 $$ \eta _{A}=\frac{m_{p}}{4\sigma _{in}}\left ( \frac{\pi \gamma}{\mu} \right )^{1/2}\frac{2\mu -1}{1-\mu} \frac{v_{A}^{2}}{\rho _{0}c_{S}} \approx 1.91\times 10^{-9} \frac{2\mu -1}{(1-\mu )\mu ^{1/2}} \frac{v_{A}^{2}}{\rho _{0}c_{S}}. $$

Since we are interested in periodic spatio-temporal changes, we write all perturbations proportional to ${\widehat{f}}(x)\exp [i(kz-\omega t)]$, where ${\widehat{f}}(x)$ is the amplitude of the perturbations, $k$ is the longitudinal wavenumber, and $\omega $ is the frequency of waves.

At this stage, we ought to make a differentiation between the fully and partially ionised cases and use the fully ionised results as a benchmark.

### Fully Ionised Case

To correctly interpret our results and evidence the effect of partial ionisation on the properties of guided waves, we are going to use the solutions obtained for a fully ionised plasma as a benchmark. When we choose $\mu =0.5$ in Equation 8, the plasma is fully ionised and the dispersion relation of waves reduces to the expression derived by Roberts (1981). In this case, the frequency of waves is a real quantity.

To make the analysis of the dispersion relation of waves along an interface easier to study, we write the dispersion relation in dimensionless form, where the frequency of waves is expressed in units of the Alfvén frequency in region 1; therefore, the eigenvalue of the problem will be $X=\omega /kv_{A1}$. Writing the density ratio $\rho _{1}/\rho _{2}=d$, due to the particular choice of the equilibrium, we have that

$$ \frac{c_{S2}}{c_{S1}}=\frac{v_{A2}}{v_{A1}}=\sqrt{d}, \quad \frac{c_{S1}}{v_{A1}}=\sqrt{\frac{\gamma \beta}{2}}, $$

with $\beta $ being the plasma-$\beta $ value, which will be identical in both regions.

The system of equations 2 – 6 can be reduced to a single second-order ordinary differential equation for the transversal component of the velocity of the form

9 $$ \frac{d^{2}{\widehat{v}}_{x}}{dx^{2}}+m_{0}^{2}{ \widehat{v}}_{x}=0, $$

where the quantity $m_{0}$ defined as

10 $$ m_{0}=\sqrt{ \frac{(\omega ^{2}-k^{2}c_{S}^{2})(\omega ^{2}-k^{2}v_{A}^{2})}{(c_{S}^{2}+v_{A}^{2})(\omega ^{2}-k^{2}c_{T}^{2})}} $$

is the magnetoacoustic parameter and plays the role of an effective wavenumber. In the above expression

$$ c_{T}^{2}=\frac{c_{S}^{2}v_{A}^{2}}{c_{S}^{2}+v_{A}^{2}} $$

is the tube speed.

As a result, the variation of the normal component of the velocity can be written as

11 $$ \widehat{v}_{x}(x) = \left \{ \textstyle\begin{array}{l@{\quad}l} \alpha _{1} e^{ m_{1} x},& x < 0, \\ \alpha _{2} e^{- m_{2} x},& x > 0,\end{array}\displaystyle \right . $$

where the magnetoacoustic parameters in the two regions can be written in dimensionless form as

$$ m_{1}=\sqrt{ \frac{( \gamma \beta - 2X^{2})(1- X^{2})}{\gamma \beta -X^{2}(\gamma \beta +2)}}, \quad m_{2}=\sqrt{ \frac{(d\gamma \beta - 2X^{2})(d-X^{2})}{d^{2}\gamma \beta -dX^{2}(\gamma \beta +2)}}. $$

In practice, the equation that describes the changes in the $x$-component of the velocity perturbation 9 can also be written in terms of the normal component of the magnetic perturbation given the simple relationship between these quantities (${\widehat{b}}_{x}=kB_{0}{\widehat{v}}_{x}/ \omega $) and the expression of the magnetoacoustic parameter would be identical.

Solutions of the governing differential equation obtained in the two regions can be joined at the $x=0$ interface by imposing the continuity of the total pressure and the normal components of the velocity, so the dimensionless form of the dispersion relation becomes

12 $$ d(1-X^{2})m_{2}+(d-X^{2})m_{1}=0, $$

which is the dispersion relation derived and studied by Roberts (1981) written in dimensionless form. Restricting our analysis to forward propagating waves, the above dispersion relation will admit solutions only if the phase speed of waves lies between the two Alfvén speeds, or –in dimensionless form– when

13 $$ \text{min}\left (1,\sqrt{d}\right )< X< \text{max}\left (1, \sqrt{d} \right ). $$

In addition, the solutions have to be evanescent in the transversal direction, which implies that the magnetoacoustic parameters in the two regions have to satisfy the conditions that $m_{1}^{2}>0$ and $m_{2}^{2}>0$, which means that the phase speed of waves has to satisfy the condition (in dimensional form)

14 $$ \frac{\omega}{k}\in [0,c_{Tj})\cup [\text{min}(c_{j},v_{Aj}), \text{max}(c_{j},v_{Aj})], \quad j=1,2. $$

In our approach, the min/max that appears in the above relation translates into conditions imposed on plasma-$\beta $. In dimensionless form, these conditions can be written as

15 $$ X\in \left \{ \textstyle\begin{array}{c@{\quad}c} \Big[0,\sqrt{\frac{\gamma \beta}{\gamma \beta +2}} \Big) \cup \left [ \sqrt{\frac{\gamma \beta}{2}},1\right ], & \beta < \frac{2}{\gamma} \approx 1.2 \\ \Big[0,\sqrt{\frac{\gamma \beta}{\gamma \beta +2}}\Big)\cup \left [1, \sqrt{\frac{\gamma \beta}{2}}\right ], & \beta >1.2 \end{array}\displaystyle \right . $$

in region 1, and

16 $$ X\in \left \{ \textstyle\begin{array}{c@{\quad}c} \Big[0, \sqrt{\frac{d\gamma \beta}{\gamma \beta +2}}\Big)\cup \left [ \sqrt{\frac{d\gamma \beta}{2}},\sqrt{d}\right ], & \beta < 1.2 \\ \Big[0,\sqrt{\frac{d\gamma \beta}{\gamma \beta +2}}\Big)\cup \left [ \sqrt{d},\sqrt{\frac{d\gamma \beta}{2}}\right ],& \beta >1.2 \end{array}\displaystyle \right . $$

in region 2. When imposing the evanescence condition on both sides of the discontinuity, the density ratio, $d$, will also be the key parameter that helps in the ordering of characteristic speeds.

When combining the above intervals and the condition 13, it is clear that there are no solutions of the dispersion relation when $\beta <1.2$ (typical for plasma environments dominated by magnetic forces). Mathematically, this can be easily proven. Let us expand the dispersion relation 12 into a series with respect to plasma-$\beta $. In this case the dispersion relation 12 reduces to

17 $$ \left (1-\frac{\gamma \beta}{4}\right )\left [ \sqrt{d}(1-X^{2})\sqrt{d-X^{2}}+(d-X^{2}) \sqrt{1-X^{2}}\right ]+{\mathcal {O}}(\beta ^{2})=0. $$

Neglecting terms ${\mathcal {O}}(\beta ^{2})$, the above expression admits as solutions the values of $X=1$ and $X=\sqrt{d}$, which are degenerate solutions of the dispersion relation, therefore these are omitted (we call *a degenerate solution* the solution that corresponds to $\omega =kv_{A}$ and $\omega =kv_{Ae}$). This result is valid for both $d<1$ and $d>1$ cases.

**Figure 1:**
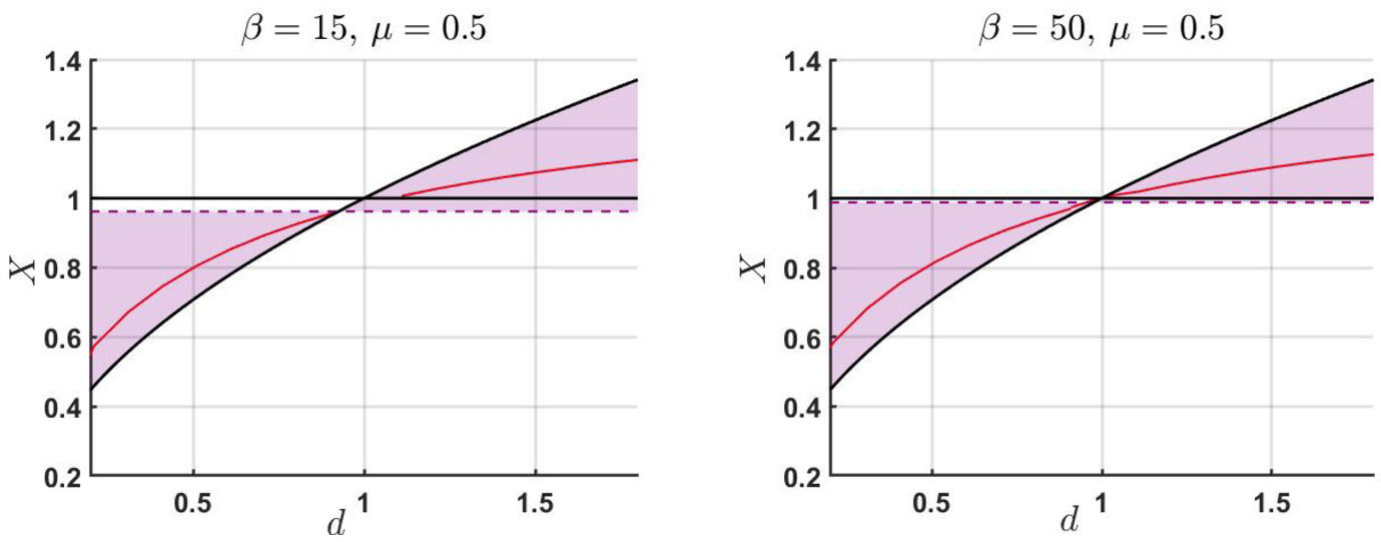
The variation of the dimensionless frequency of waves $X$ in terms of the density ratio $d$ when $\beta =15$ (left panel) and $\beta =50$ (right panel). The colored region corresponds to the location in the parametric space where solutions are allowed.

The numerical solutions of the dispersion relation 12 are obtained using the Newton-Raphson method, and these are represented as the variation of the dimensionless frequency of waves in terms of the density ratio, $d$, for two distinct values of plasma-$\beta $. Our findings are shown in Figure 1.

When $\beta >1.2$ (the plasma dynamics is driven by pressure forces), waves propagating with phase speed close to the Alfvén speed in the parallel direction to the ambient magnetic field are slow magnetoacoustic waves.

The regions where possible solutions are expected are shown as the overlap between the condition 13, with boundaries shown by thick solid black lines, and the corresponding conditions 15 – 16, shown here as regions bounded by dashed lines. Although the pairs of ($d$, $X$) that correspond to the two solid black lines are mathematical solutions, physically they are not accepted, as these are degenerate solutions of the dispersion relation. The physically accepted solutions are shown by red lines. The frequency of waves (or their phase speed) increases with the density ratio between the two regions.

Inspired by the graphical representation of the solutions of the dispersion relation, we can now derive an approximate analytical solution of the dispersion relation. It is clear that as long as $d<1$ the dependence of $X$ on the density ratio, $d$, is such that $X^{2}\approx d+\delta $, where $\delta $ is a small and positive quantity. Inserting this expression back into the dispersion relation 12, we obtain that

$$ \delta =\frac{d}{2} \frac{(\gamma \beta -2)(1-d)[\gamma \beta -d(\gamma \beta +2)]}{\gamma \beta -2d}. $$

Therefore, the equation that describes the forward propagating wave becomes

18 $$ X\approx \sqrt{d}\left \{1+\frac{1}{2} \frac{(\gamma \beta -2)(1-d)[\gamma \beta -d(\gamma \beta +2)]}{\gamma \beta -2d} \right \}^{1/2}. $$

Imposing the condition that $\delta $ remains a positive quantity means that the condition $d<\gamma \beta /(\gamma \beta +2)$ has to be satisfied, so solutions in this regime are bounded by the value of the density contrast between the two regions.

On the other hand, if $d>1$, then we can write that $X^{2}\approx 1+\epsilon $, where $\epsilon $ is, again, a small and positive quantity. After a straightforward calculation, it is easy to obtain that

$$ \epsilon = \frac{(d-1)(\gamma \beta -2)(d\gamma \beta -\gamma \beta -2)}{2d(d\gamma \beta -2)}, $$

which means that the equation describing the forward propagating wave is given by

19 $$ X\approx \left \{1+ \frac{(d-1)(\gamma \beta -2)(d\gamma \beta -\gamma \beta -2)}{2d(d\gamma \beta -2)} \right \}^{1/2}. $$

A simple analysis reveals that $\epsilon $ will be a positive quantity provided the condition $d>(\gamma \beta +2)/\gamma \beta $ is satisfied. The two conditions imposed on the small parameters $\delta $ and $\epsilon $ explain why solutions for $X$ are not found near the value of 1 in the left panel of Figure 1.

### Partially Ionised Case

Given the partially ionised character of the plasma ($\mu \neq 0.5$) and the presence of the ambipolar diffusion, the frequency of waves is determined by the real part of $\omega $, while the damping rate of waves is determined by the imaginary part that, for the particular ansatz we used here, has to be a negative quantity. In what follows, we are going to drop the *hat* symbol.

The system of MHD equations 2 – 6 can be reduced to a system of coupled ordinary differential equations of the form

20 $$ \frac{\omega ^{2}c_{S}^{2}}{\omega ^{2}-k^{2}c_{S}^{2}} \frac{d^{2} v_{x}}{dx^{2}}+\omega ^{2}v_{x}+ \frac{\omega k B_{0}}{\mu \rho _{0}}b_{x}+ \frac{i\omega B_{0}}{\mu \rho _{0}}\frac{db_{z}}{dx}=0, $$

21 $$ \left (\omega +i\eta _{A} k^{2}-i\eta _{A} \frac{d^{2}}{d x^{2}} \right )b_{x}=-kB_{0}v_{x}, $$

22 $$ \left (\omega +i\eta _{A} k^{2}-i\eta _{A} \frac{d^{2}}{d x^{2}} \right )b_{z}=-iB_{0}\frac{d v_{x}}{d x}, $$

23 $$ \frac{db_{x}}{dx}+ikb_{z}=0. $$

After a straightforward set of algebraic manipulations, the above system of equations can be reduced to a differential equation that describes the variation of the $x$-component of the magnetic field perturbation

24 $$ A\frac{d^{4}b_{x}}{dx^{4}}+B \frac{d^{2}b_{x}}{dx^{2}}+Cb_{x}=0, $$

where the coefficients of this equation are given by

$$ A=\omega c_{S}^{2}\eta _{A}, \quad B=iv_{A}^{2}(\omega ^{2}-k^{2}c_{S}^{2})+ \omega \left [\omega ^{2}\eta _{A}+c_{S}^{2}(i\omega -2k^{2}\eta _{A}) \right ], $$

$$ C=i(\omega ^{2}-k^{2}c_{S}^{2})\left [\omega (\omega +ik^{2}\eta _{A})-k^{2}v_{A}^{2} \right ]. $$

We should note here that in a fully ionised plasma, the above differential equation reduces to a quadratic form

25 $$ \frac{d^{2}b_{x}}{dx^{2}}+m_{0}^{2}b_{x}=0, $$

where the magnetoacoustic parameter, $m_{0}$, was defined earlier by Equation 10. Although in the original study by Roberts (1981) the governing equation similar to Equation 25 is written for the transversal component of the velocity, $v_{x}$, thanks to a simple linear relationship between $v_{x}$ and $b_{x}$ given earlier, it can be shown that the two formulations are equivalent.

In addition to the dimensionless quantities employed earlier, we introduce the quantity $Q=\eta _{A}k/v_{A}$ to denote the dimensionless dissipative coefficient. As a result, the coefficients of the governing equation 24 can be written as

$$ A=X\frac{\gamma \beta}{2}Q, \quad B=k^{2}\left \{i\left (X^{2}- \frac{\gamma \beta}{2}\right )+X\left [X^{2}Q+\frac{\gamma \beta}{2} \left (iX-2Q\right )\right ]\right \}, $$

26 $$ C=ik^{4}\left (X^{2}-\frac{\gamma \beta}{2}\right )[X(X+iQ)-1]. $$

The quantity $Q$ introduced above would incorporate all the information about the partially ionised nature of the plasma and will play a crucial role in the description of the wave damping. In terms of the dimensionless variables introduced earlier, the quantity $Q$ in the two plasma regions can be written as

$$ Q_{1}=\frac{m_{p}}{4\sigma _{in}}\left (\frac{2\pi}{\beta \mu}\right )^{1/2} \frac{2\mu -1}{1-\mu}\frac{k}{\rho _{01}}, $$

and

$$ Q_{2}=\frac{m_{p}}{4\sigma _{in}}d^{2}\left ( \frac{2\pi d}{\beta \mu }\right )^{1/2} \frac{2\mu -1}{d_{n}\mu -(2\mu -1)d}\left [2\mu -(2\mu -1) \frac{d}{d_{n}}\right ]^{1/2}\frac{k}{\rho _{01}}, $$

where $d_{n}=\rho _{n1}/\rho _{n2}$. The expressions of $Q_{1}$ and $Q_{2}$ depend on the density of the medium and the wavenumber. We use $\rho _{01}=5\times 10^{-9}\text{ kg}\text{ m}^{-3}$ and $k=5\times 10^{-6}\text{ m}^{-1}$ as representative values in region 1. Since we expect $Q_{2}$ to be a real and positive quantity, the restriction that needs to be imposed is that $d_{n}>(2\mu -1)d/\mu $.

Assuming a trial solution of the form proportional to $e^{mx}$, the auxiliary equation reduces to a bi-quadratic equation whose roots are simply

27 $$ m_{1,2}= \sqrt{-\frac{B}{2A}\pm \frac{1}{2A}\sqrt{B^{2}-4AC}}, $$

and

28 $$ m_{3,4}= -\sqrt{-\frac{B}{2A}\pm \frac{1}{2A}\sqrt{B^{2}-4AC}}. $$

A similar equation has to be solved on both sides of the interface; however, to ensure the evanescence of solutions, we write

29 $$ b_{x} =\left \{ \textstyle\begin{array}{c@{\quad}c} \alpha _{1} e^{m_{1}x}+\alpha _{2} e^{m_{2}x}, & x < 0, \\ \alpha _{3} e^{m_{3} x}+\alpha _{4} e^{m_{4} x}, & x > 0, \end{array}\displaystyle \right . $$

where the quantities $m_{1}$ and $m_{2}$ are given in terms of the coefficients in Equation 26 (with $Q=Q_{1}$), while for $m_{3}$ and $m_{4}$ we use

30 $$\begin{gathered} A=X\frac{d\gamma \beta}{2}Q_{2}, \quad B=k^{2}d\left \{i\left (X^{2}- \frac{d\gamma \beta}{2}\right )+X\left [X^{2}Q_{2}+ \frac{d\gamma \beta}{2}\left (iX-2Q_{2}\right )\right ]\right \}, \\ C=ik^{4}\left (X^{2}-\frac{d\gamma \beta}{2}\right )[X(X+iQ_{2})-d]. \end{gathered}$$

The constant coefficients $\alpha _{1}-\alpha _{4}$ in Equation 29 can be determined upon applying boundary conditions at the interface.

In a fully ionised and ideal plasma, the boundary conditions (also known as jump conditions) are determined by imposing the continuity of transversal components of the Maxwell-Reynolds stress tensor, which translates into the continuity of the transversal component of the velocity and the total pressure. However, in a partially ionised plasma, the presence of neutrals generates electric currents perpendicular to the ambient magnetic field (dissipated via ambipolar resistivity), and the magnetic topology can change across the interface, and as a result, magnetic field lines can diffuse across the interface, perturbing its stability. The jump conditions in this case can be obtained in a similar fashion as in the study by Díaz, Khomenko, and Collados (2014), i.e. we derive the jump conditions using the integral form of conservation laws applied to an infinitesimally thin region surrounding the boundary. As a result, the jump conditions that are applied at the boundary between two partially ionised plasmas situated at $x=0$ in the presence of ambipolar diffusion can be written as

$$ \bigr[\bigr[ \rho _{0}c_{S}^{2}v_{x}\bigr]\bigr]=0, $$

$$ \Bigr[\Bigr[ p+\frac{B_{0}b_{z}}{\mu}\Bigr]\Bigr]=0, $$

$$ \Bigr[\Bigr[ \eta _{A}\left (\frac{db_{z}}{dx}-ikb_{x}\right )-B_{0}v_{x} \Bigr]\Bigr]=0, $$

31 $$ \bigr[\bigr[ \eta _{A}b_{z}\bigr]\bigr]=0, $$

where $\bigr[\bigr[ Z\bigr]\bigr]=\lim _{x\to 0^{+}}[Z(x)-Z(-x)]$ denotes the jump in the quantity $Z$ across the interface. Although the first two jump conditions are similar to their fully ionised counterparts, the expressions of the normal component of velocity and total pressure will contain information about the ambipolar diffusion, encapsulating the partially ionised character of the plasma.

Due to the particular equilibrium configuration of our model, the first jump condition reduces to the continuity of the transversal component of the velocity vector, the second one denotes the continuity of the total pressure, while the last two jump conditions originate from the components of the induction equation. In a fully ionised plasma ($\eta _{A}=0)$, the last two relations become redundant.

The MHD equations can be reduced so that physical variables that appear in the above boundary conditions can all be written in terms of the normal component of magnetic field perturbation, $b_{x}$. As a result, the jump conditions transform into

$$\begin{gathered} \Bigr[\Bigr[ \left (\omega +i\eta _{A}k^{2}-i\eta _{A} \frac{d^{2}}{dx^{2}}\right )b_{x}\Bigr]\Bigr]=0,\\ \Bigr[\Bigr[ \frac{\rho _{0}}{\omega ^{2}-k^{2}c_{S}^{2}}\left [ \omega c_{S}^{2}(\omega +i\eta _{A}k^{2})+v_{A}^{2}(\omega ^{2}-k^{2}c_{S}^{2}) \right ]\frac{db_{x}}{dx}- \frac{i\rho _{0}\omega c_{S}^{2}\eta _{A}}{\omega ^{2}-k^{2}c_{S}^{2}} \frac{d^{3}b_{x}}{dx^{3}}\Bigr]\Bigr]=0, \end{gathered}$$

$$ \Bigr[\Bigr[\left (\omega +i\eta _{A}k^{2}-2i\eta _{A} \frac{d^{2}}{dx^{2}}\right )b_{x}\Bigr]\Bigr]=0, $$

32 $$ \Bigr[\Bigr[\eta _{A}\frac{db_{x}}{dx}\Bigr]\Bigr]=0. $$

Using the form of the normal component of the magnetic field given by Equation 29, the four jump conditions can be written as a system of equations for the 4 constants that appear in that equation.

Using the proposed form of $b_{x}$ on both sides of the interface, the jump conditions lead to a system of equations for the coefficients $\alpha _{j}$ of the form

33 $$ {\mathcal {M}}_{ij}\alpha _{j}^{T}=0, $$

where $\alpha _{j}^{T}$ is the transpose matrix of the unknown quantities $\alpha _{j}$ and the elements of the $4\times 4$ matrix ℳ are given in the Appendix. The condition of non-trivial solutions of the above homogeneous system of equations can be written as

34 $$ \det ({\mathcal {M}}_{ij})=0, $$

which constitutes the dispersion relation of the problem that will be solved numerically.

## Numerical Solutions

With the solutions of the dispersion relation in the case of fully ionised plasma determined earlier, we can turn our attention to the case of surface waves in partially ionised plasma. In general, the solutions of the dispersion relation 34 are complex quantities, and finding the solution to this equation analytically is still difficult as the equation is highly transcendental.

Let us first discuss the physical domains where surface waves are allowed to propagate. First of all, the condition of evanescence in this case is defined differently. Although qualitatively the $x$-dependence of the normal component of the magnetic field perturbation given by Equation 29 is similar to the one we obtained in the case of fully ionised plasma, now the magnetoacoustic parameters are complex quantities, therefore the evanescence of waves in the lateral direction would imply a decaying oscillatory behaviour. To achieve this, we need to impose that the real parts of the ${ m}_{j}$ parameters are positive. This restriction will be achieved by limiting our analysis to the case when the arguments of the complex parameters ${m}_{j}$ are in the interval $-\pi /2\leq \text{arg}({m}_{j})\leq \pi /2$.

**Figure 2:**
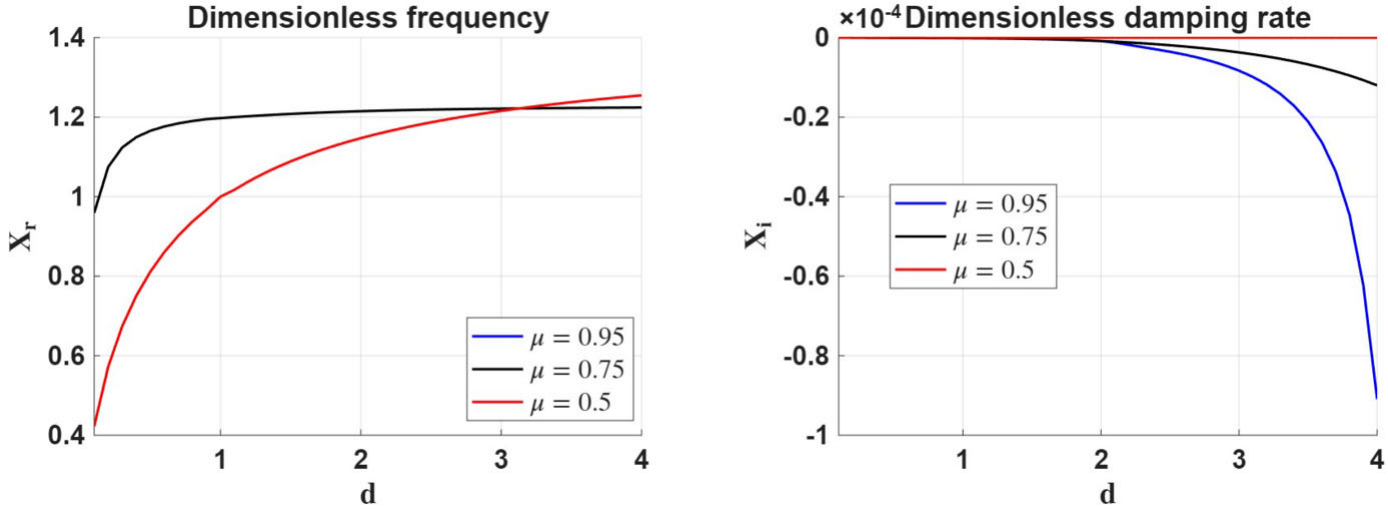
The variation of the real and imaginary part of the dimensionless frequency, $X$, in terms of the density ratio between the two regions for various values of the ionisation degree. These plots were obtained considering $\beta =50$ and $d_{n}=4$.

Figure 2 shows the variation of the real (left panel) and imaginary (right panel) part of the variable $X$ in terms of the density ratio, $d$, for the chosen plasma parameters for three distinct values of the ionisation degree covering a large spectrum of values. In the way the variable $X$ is defined, the imaginary part describes the damping rate in Alfvén frequency units. The two figures were obtained for the particular values of $\beta =50$ and $d_{n}=4$. The three curves were plotted for three different ionisation degrees, where $\mu =0.5$ corresponds to a fully ionised plasma, $\mu =0.75$ represents a moderate ionisation, and $\mu =0.95$ corresponds to a weakly ionised plasma condition, respectively.

The left panel of Figure 2 demonstrates that, across all ionisation degrees, the phase speed of the surface waves increases monotonically with the density contrast, $d$. This trend closely follows the behaviour established in the fully ionised reference case (see Figure 1), suggesting that the presence of neutrals and ambipolar diffusion does not significantly alter the wave propagation speed. This result is somewhat surprising, as one might intuitively expect the additional neutral inertia to reduce the phase speed. However, due to the high plasma-$\beta $ regime considered here, the thermal pressure dominates over magnetic tension, and wave dynamics are primarily governed by compressive effects. In such conditions, ambipolar diffusion weakens the coupling between ions and neutrals, allowing ions to support the wave motion more independently. This results in a weak dependence of $X_{r}$ on the ionisation degree, as clearly seen in the overlapping curves. At $d\approx 3$, a crossover takes place, when for $d>3$ the frequency of surface waves in a fully ionised plasma becomes larger than the corresponding frequency in the case of partial ionisation. One possible explanation of this phenomenon is that when $d<3$ in a partially ionised plasma, the interface is less asymmetric, so wave energy is more evenly distributed between the two regions. Ambipolar diffusion decouples neutrals from the magnetic field, allowing ions to move more freely. This results in a lower effective inertia for the ions, despite the total mass being higher. With less inertia and weaker magnetic tension, the wave becomes faster, hence higher $x_{r}$ than in the fully ionised case. In contrast, in a fully ionised case, ions and the magnetic field are tightly coupled, and the surface wave carries all the plasma mass coupled to the field, leading to slightly lower phase speed (lower $X_{r}$). When $d>3$, the interface becomes highly asymmetric, i.e. region 1 is much denser than region 2. In the fully ionised case, the wave becomes increasingly confined to the denser region, where the Alfvén speed is lower due to higher density. However, the restoring magnetic tension is more effective because ions and the magnetic field are fully coupled. In the partially ionised case, as $d$ increases, the denser region contains more neutrals, which are decoupled from the field, contributing inertia but not tension. Therefore, the effective restoring force saturates, but inertia keeps growing, resulting in waves being slowed down, so $X_{r}$ becomes smaller than the fully ionised values.

The right panel shows that, although damping remains weak overall (order $10^{-5}-10^{-4}$), it exhibits a clear dependence on ionisation degree. The damping rate increases significantly as the plasma becomes more neutral, with the $\mu =0.95$ case showing the strongest attenuation. This reflects the enhanced role of ambipolar diffusion in weakly ionised plasmas, where ion–neutral decoupling leads to stronger frictional dissipation. Furthermore, damping becomes slightly more pronounced with increasing density contrast $d$, which means that waves undergo a stronger confinement near the interface, where the gradients in ambipolar diffusion are enhanced.

**Figure 3:**
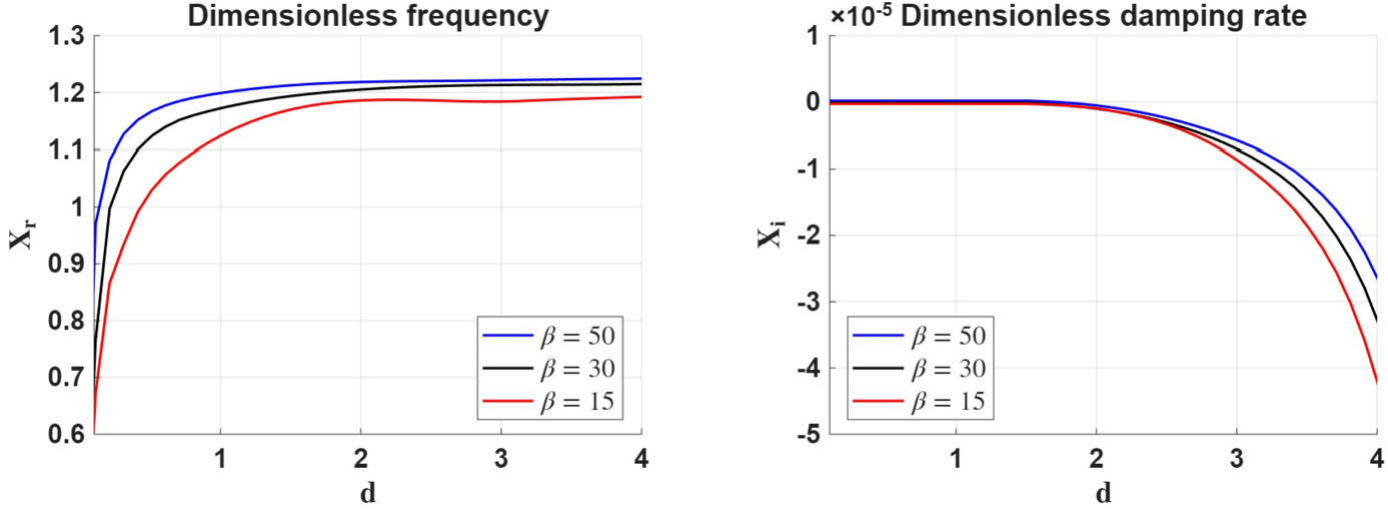
The same as in Figure 2, but here we consider the variation of the real and imaginary parts of $X$ in terms of the density ratio, $d$, for various values of $\beta $, considering $d_{n}=4$ and $\mu =0.85$.

Figure 3 displays the variation of the real (left panel) and imaginary (right panel) parts of the dimensionless frequency of waves propagating along the density interface in terms of the density ratio, for three values of plasma-$\beta $ for a given value of $d_{n}=4$ and an ionisation degree of the plasma of $\mu =0.85$. While the propagation speed of waves changes very little with plasma-$\beta $ (left panel), the damping rate of surface waves propagating along the interface shows higher values for a larger density ratio. The dependence of $X_{r}$ on plasma-$\beta $ is in line with the influence of thermal pressure versus magnetic tension. In high plasma-$\beta $ plasmas, pressure gradients dominate wave dynamics, allowing faster propagation. As $\beta $ decreases, magnetic effects become more pronounced, and the coupling between ions and neutrals becomes tighter, which acts to slow down the wave.

The right-hand panel shows that damping remains weak but increases with decreasing plasma-$\beta $. In lower-$\beta $ conditions, stronger magnetic coupling leads to more effective ion–neutral friction and hence greater energy dissipation. In contrast, high-$\beta $ plasmas favour thermal pressure dominance and allow ion–neutral decoupling, reducing the damping efficiency of ambipolar diffusion. Irrespective of the value of plasma-$\beta $, the damping rate of surface waves increases with the density ratio, $d$.

**Figure 4:**
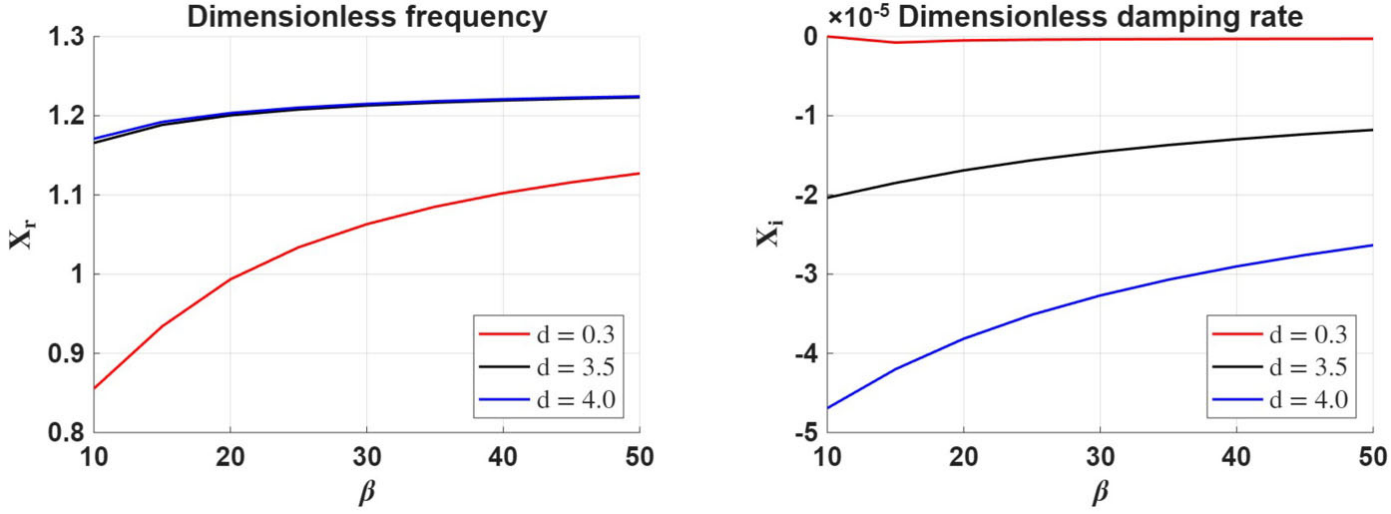
The same as in Figure 2, but here we consider the variation of the real and imaginary part of $X$ in terms of plasma-$\beta $ for various values of the density ratio, $d$, considering $d_{n}=4$ and $\mu =0.85$.

The variation of the real and imaginary parts of the variable $X$ with respect to the plasma-$\beta $ parameter for a given density ratio, $d$, ionisation degree and $d_{n}$ is shown in Figure 4. First of all, the phase speed of waves shown in the left-hand side panel increases with the density ratio (as established earlier), and the phase speed of waves increases with the plasma-$\beta $. With the increase of plasma-$\beta $, the phase speed of waves tends to saturate, suggesting that further increases in plasma pressure have a negligible effect on the phase speed. In the high plasma-$\beta $ regime, ion–neutral decoupling becomes important, reducing the impact of neutral drag. As a result, the wave behaves more like an acoustic surface wave, allowing faster propagation.

The right-hand side panel of Figure 4 shows that the damping rate of waves decreases with the value of plasma-$\beta $, meaning that waves are more weakly damped in high plasma-$\beta $ regimes. With an increase of $\beta $, the ions decouple more effectively from the neutrals, and wave dynamics become dominated by pressure forces. As a result, fewer energy losses occur through ion–neutral friction, and the wave damping rate decreases. The damping rate increases with the density ratio between the two regions, meaning that higher density contrast implies larger asymmetry in ambipolar diffusion across the interface, favouring more energy dissipation near the denser side.

**Figure 5:**
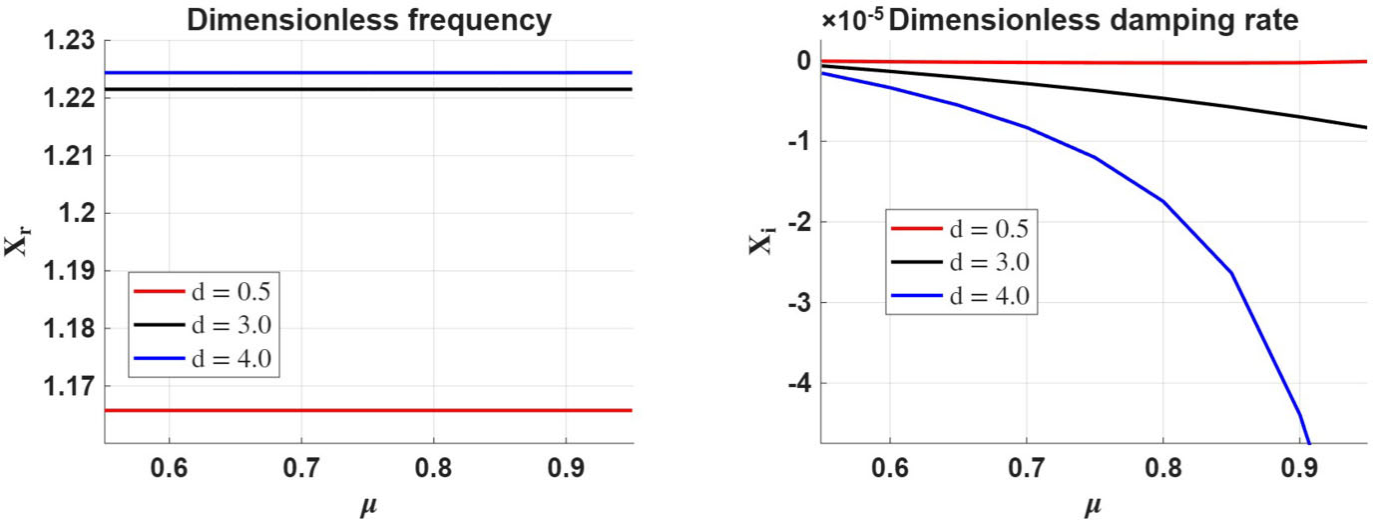
The same as in Figure 2, but here we study the variation of the real and imaginary part of $X$ in terms of the ionisation degree of the plasma ($\mu $) for various values of the density ratio, $d$, considering $d_{n}=4$ and $\beta =50$.

When we vary the ionisation degree of the plasma and keep the plasma-$\beta$ and $d_{n}$ constant (see Figure 5), the propagation speed (frequency) of waves propagating at a density interface separating two regions of different ionisation degree practically does not depend on the ionisation degree of the plasma and, as before, their speed increases with the density ratio of the two regions. In the high plasma-$\beta $ regime, kinetic pressure forces dominate, and magnetic tension is weak. In this case, even with the amount of neutrals increasing, ambipolar diffusion reduces ion–neutral coupling, allowing ions to respond more freely to wave motion. As a result, the presence of neutrals does not significantly affect the waves’ ability to propagate and, consequently, their phase speed.

In contrast, the imaginary part of the variable $X$, showing the damping rate of waves (right panel), shows a rather strong variation with the ionisation degree. Clearly, for an ionisation degree close to the value corresponding to a fully ionised plasma, the damping rate is very small, regardless of the density ratio, $d$. As the neutral fraction increases, ambipolar diffusion becomes more significant, leading to enhanced wave damping. This effect is most pronounced when the density contrast between regions is high, amplifying asymmetries in ambipolar resistivity across the interface.

## Conclusion

Our analysis has focused on the properties of waves propagating at an interface separating two regions of partially ionised plasmas, permeated by a magnetic field that is parallel to the discontinuity. For simplicity, we considered that the two regions have the same equilibrium pressure and magnetic field; therefore, the discontinuity appears as a jump in equilibrium density, and the strength of this jump constitutes one of the key parameters that influence the properties of waves. This equilibrium model also means that our analysis is valid for a constant plasma-$\beta $ parameter. Since we considered that the collisional frequency between particles is much larger than the frequency of waves, the dynamics was described within a single-fluid framework, where the partially ionised effects appear through the generalised Ohm’s law and the imperfect coupling of neutral and charged particles (ions) generates the ambipolar diffusion.

Using a normal mode analysis, we derived the dispersion relation of waves. The presence of ambipolar diffusion in the governing equations renders the frequency of waves a complex quantity, where the imaginary part describes the damping of waves. The earlier results obtained by Roberts (1981) for a fully ionised plasma were used as a reference to highlight the effect of neutrals on wave propagation and damping. First of all, a thorough analysis of the results by Roberts (1981) revealed that the model we employed allows the propagation of waves only when $\beta >2/\gamma \approx 1.2$, i.e. when the dynamics is driven by pressure forces, a case that is relevant to photospheric structures with moderate strength of the magnetic field.

Similar to waves in a fully ionised case, the frequency (or phase speed) of waves increases with the density contrast between the two regions. Interestingly, the wave frequency remains nearly unchanged with varying ionisation degree, indicating that ambipolar diffusion primarily affects wave damping, not propagation. The damping of waves is primarily governed by the presence of neutrals and the value of the plasma-$\beta $ parameter. In weakly ionised plasmas, ambipolar diffusion enhances frictional losses through ion–neutral decoupling, leading to stronger damping. Conversely, higher $\beta $ values favour pressure-driven dynamics and reduce magnetic coupling, making ambipolar damping less effective. While the surface waves studied here are compressible and arise from a discontinuity in plasma density, unlike the incompressible Alfvén waves typically discussed in uniform partially ionised plasmas, the underlying dissipative mechanism due to ambipolar diffusion is shared. This naturally suggests a qualitative analogy with the damping behaviour described in, e.g., Soler (2024), where increasing wavenumber leads to overdamped and purely damped Alfvén waves. Although our surface wave geometry is distinct, a detailed study of the wavenumber dependence could determine whether similar transitions occur, and such an investigation is left for future work.

The techniques we introduced and the methodology used to determine dispersion relations will help us to transpose these ideas to the problem of wave propagation in a partially ionised plasma slab waveguide that will be studied in the near future.

## Data Availability

No datasets were generated or analysed during the current study.

## Appendix: The Elements of the Matrix ℳ in Equation 33

The matrix elements in the matrix equation 33 are given as

$$ {\mathcal {M}}_{11}=X+iQ_{1}-\frac{iQ_{1}}{k^{2}}m_{1}^{2}, \quad { \mathcal {M}}_{12}=X+iQ_{1}-\frac{iQ_{1}}{k^{2}}m_{2}^{2}, $$

$$ {\mathcal {M}}_{13}=-X-iQ_{2}+\frac{iQ_{2}}{k^{2}}m_{3}^{2},\quad { \mathcal {M}}_{14}=-X-iQ_{2}+\frac{iQ_{2}}{k^{2}}m_{4}^{2}, $$

$$ {\mathcal {M}}_{21}=\left [\frac{X\gamma \beta}{2X^{2}-\gamma \beta}(X+iQ_{1})+1 \right ]m_{1}-\frac{iXQ_{1}\gamma \beta}{k^{2}(2X^{2}-\gamma \beta )}m_{1}^{3}, $$

$$ {\mathcal {M}}_{22}=\left [\frac{X\gamma \beta}{2X^{2}-\gamma \beta}(X+iQ_{1})+1 \right ]m_{2}-\frac{iXQ_{1}\gamma \beta}{k^{2}(2X^{2}-\gamma \beta )}m_{2}^{3}, $$

$$ {\mathcal {M}}_{23}=\left [-\frac{X\gamma \beta}{2X^{2}-\gamma \beta d}(X+iQ_{2})-1 \right ]m_{3}+ \frac{iXQ_{2}\gamma \beta}{k^{2}(2X^{2}-\gamma \beta d)}m_{3}^{3}, $$

$$ {\mathcal {M}}_{24}=\left [-\frac{X\gamma \beta}{2X^{2}-\gamma \beta d}(X+iQ_{2})-1 \right ]m_{4}+ \frac{iXQ_{2}\gamma \beta}{k^{2}(2X^{2}-\gamma \beta d)}m_{4}^{3}, $$

$$ {\mathcal {M}}_{31}={\mathcal {M}}_{32}=1,\quad {\mathcal {M}}_{31}={ \mathcal {M}}_{32}=-1, $$

$$ {\mathcal {M}}_{41}=Q_{1}m_{1}, \quad {\mathcal {M}}_{42}=Q_{1}m_{2}, \quad {\mathcal {M}}_{43}=-Q_{2}m_{3}, \quad {\mathcal {M}}_{44}=-Q_{2}m_{4}. $$

Although these coefficients are aimed to be written in dimensionless form, we had to keep the $k$-dependence in the denominators of some terms as the quantities $m_{j}$ are proportional to $k$.
